# The Impact of Age-Related Stereotypes on Risky Decision-Making in the Balloon Analogue Risk Task

**DOI:** 10.1093/geroni/igaf122.1401

**Published:** 2025-12-31

**Authors:** Shuyao Liao, Yi Lu, Gu Ma, Zheng Guo, Yatian Zhou, Yue Yang Sun, Tianyuan Li, Xin Zhang

**Affiliations:** Peking University, Beijing, Beijing, China (People’s Republic); Cornell University, Ithaca, New York, United States; Peking University, Beijing, Beijing, China (People’s Republic); Peking University, Beijing, Beijing, China (People’s Republic); The Chinese University of Hong Kong, Shenzhen, Shenzhen, Guangdong, China (People’s Republic); The Chinese University of Hong Kong, Shenzhen, Shenzhen, Guangdong, China (People’s Republic); The Chinese University of Hong Kong, Shenzhen, Shenzhen, Guangdong, China (People’s Republic); Peking University, Beijing, Beijing, China (People’s Republic)

## Abstract

Previous research has suggested that older adults are more loss-averse in risky decisions, and their performance can be susceptible to age-related stereotype threats. However, prior findings have predominantly focused on negative stereotypes, and the underlying cognitive mechanisms remain unclear. This study expands the literature by examining the impact of both positive and negative age-related stereotypes and its cognitive underpinnings in the Balloon Analogue Risk Task (BART), a well-established measure of experience-based risky decision-making. We randomly assigned 159 older adults (M = 67.09, SD = 5.22) to receive information containing either positive or negative age-related stereotypes, or the same neutral information as 53 younger adults (M = 20.55, SD = 2.04) received. Participants completed the BART by pumping virtual balloons. Computational models were applied to decompose the underlying cognitive dimensions influencing decision behaviors. Consistent with prior findings, modeling results revealed that compared to younger adults, older adults exhibited lower learning rates and greater loss aversion. Contrary to our expectations, further analyses suggested that age-related stereotypes impacted primarily on the prior belief of risk. Older adults exposed to positive stereotypes held lower prior beliefs of risk compared to their counterparts receiving negative stereotypes, while there was no significant priming effect of age-related stereotypes on loss aversion. Our findings elucidate age differences in the cognitive underpinnings of risky decisions in the BART and underscore that age-related stereotypes impact older adults’ decision performance by distinctively influencing their prior beliefs about risk instead of their risk or loss preferences.

